# Tissue-Specific and Ubiquitous Expression Patterns from Alternative Promoters of Human Genes

**DOI:** 10.1371/journal.pone.0012274

**Published:** 2010-08-18

**Authors:** Edwin Jacox, Valer Gotea, Ivan Ovcharenko, Laura Elnitski

**Affiliations:** 1 National Human Genome Research Institute, National Institutes of Health, Rockville, Maryland, United States of America; 2 National Center for Biotechnology Information, National Institutes of Health, Bethesda, Maryland, United States of America; Baylor College of Medicine, United States of America

## Abstract

**Background:**

Transcriptome diversity provides the key to cellular identity. One important contribution to expression diversity is the use of alternative promoters, which creates mRNA isoforms by expanding the choice of transcription initiation sites of a gene. The proximity of the basal promoter to the transcription initiation site enables prediction of a promoter's location based on the gene annotations. We show that annotation of alternative promoters regulating expression of transcripts with distinct first exons enables a novel methodology to quantify expression levels and tissue specificity of mRNA isoforms.

**Principal Findings:**

The use of distinct alternative first exons in 3,296 genes was examined using exon-microarray data from 11 human tissues. Comparing two transcripts from each gene we found that the activity of alternative promoters (i.e., P1 and P2) was not correlated through tissue specificity or level of expression. Furthermore neither P1 nor P2 conferred any bias for tissue-specific or ubiquitous expression. Genes associated with specific diseases produced transcripts whose limited expression patterns were consistent with the tissue affected in disease. Notably, genes that were historically designated as tissue-specific or housekeeping had alternative isoforms that showed differential expression. Furthermore, only a small number of alternative promoters showed expression exclusive to a single tissue indicating that “tissue preference” provides a better description of promoter activity than tissue specificity. When compared to gene expression data in public databases, as few as 22% of the genes had detailed information for more than one isoform, whereas the remainder collapsed the expression patterns from individual transcripts into one profile.

**Conclusions:**

We describe a computational pipeline that uses microarray data to assess the level of expression and breadth of tissue profiles for transcripts with distinct first exons regulated by alternative promoters. We conclude that alternative promoters provide individualized regulation that is confirmed through expression levels, tissue preference and chromatin modifications. Although the selective use of alternative promoters often goes uncharacterized in gene expression analyses, transcripts produced in this manner make unique contributions to the cell that requires further exploration.

## Introduction

The identity of a cell is largely a consequence of the genes transcribed within it. Collectively, these units define the transcriptome, which includes RNA generated from protein-coding and noncoding genes. As a primary determinant of transcriptional activation, promoters of genes position the RNA Pol II molecule at their transcription start sites (TSSs). The position of any TSS is defined in the genome by the position of the first nucleotide in the RNA transcript, also known as the +1 position. This genomic coordinate supplies additional information regarding the position of the core promoter, which lies both adjacent to and overlapping the TSS (i.e., typically from positions −50 to +50) [1]. Promoters confer activation in “*cis*”, by acting linearly along the genomic sequence, and therefore regulate the transcription of exons that are located in downstream positions. Over half of human genes contain more than one promoter [2]–[5], which are collectively described as alternative promoters. Each promoter flanks the TSS of a transcript and regulates transcription of exons falling downstream of its genomic position, which may be within an intron of the locus.

Alternative promoters provide transcript diversity and confer dimensional complexity to a cell [6]. For example, individual promoters can be selectively utilized to provide tissue-specific expression, to incorporate distinct 5′ exons into transcripts, or to change the length or content of the open reading frame [7], as illustrated in the downstream transcript of the *CD5* gene, which excludes a membrane-binding domain [8]. Notably, a substantial number of alternative promoters have no sequence conservation because they evolved independently among species [9].

Although promoter activity cannot be quantitatively addressed by DNA sequence alone, transcript expression levels serve as a proxy for evaluating promoter function. One technique for measuring transcript expression levels is CAGE (cap-analysis of gene expression), providing information about TSS location and transcript abundance. The CAGE technique has been used to assess a collection of 5′ initiation sites in 17 normal tissues [3]; however, like any resource for TSSs, CAGE data must be combined with annotated gene structures to fully elucidate the relationship between regulatory elements and transcriptional products.

Traditional cDNA or oligonucleotide microarrays represent a second extensively used method to measure transcript expression levels. However, these platforms do not address the alternative promoter activity because many isoforms share the 3′ exons that hybridize to the array probes [10]. Advances in microarray technology have enabled exon-specific microarray platforms, in which all known and predicted exons in the genome have hybridization probes. These platforms are most often used to assess alternative splicing among transcripts [11]. Using the exon microarray platform, we describe a novel approach to study alternative promoter activity using hybridization probes that anneal to first exons of genes, which enables us to evaluate the exclusive activity of the alternative promoters located upstream of these exons. Using a computational assessment of exon-array expression data, we identified and characterized a sizeable set of alternative promoters and discriminated the expression levels and tissue profiles of their transcript isoforms. Although this approach does not identify the precise boundaries of alternative promoters, the measurement of the hybridization signal associated with the first exon enables direct interpretation of the promoter-associated activity in multiple tissues. We quantified and visualized expression data for the subset of human genes containing these distinct alternative first exons (DAlFEs) to elucidate details of the “promoterome” regulating human gene expression and distinguish among alternative promoters whose mutation may contribute to disease phenotypes.

## Results

### Genome-wide analysis of alternative promoters and alternative first exons

**Figure 1:**
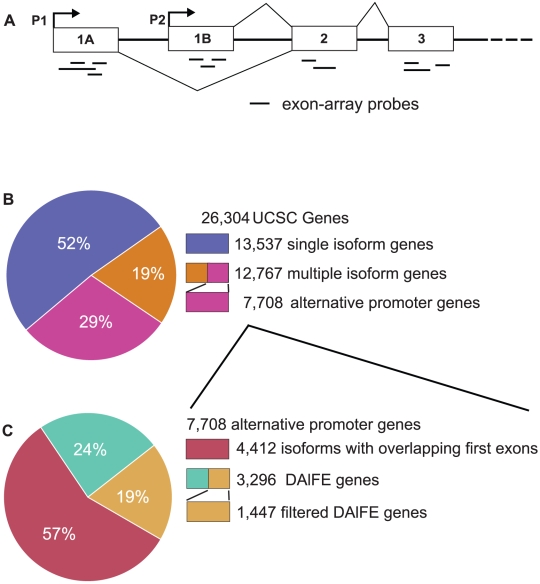
Illustration of alternative promoters and their prevalence in the human genome. (A) Isoforms containing distinct first exons (labeled 1A and 1B, respectively) are regulated by alternative promoters, P1 or P2, which lie immediately upstream of the transcription-start sites for exons 1A and 1B. These isoforms frequently share the remaining exons (2, 3, etc.). P1 represents the most upstream promoter region. It is intergenic and not transcribed. In contrast, P2 represents the intragenic (or downstream) promoter, located in a genomic region that lies in an intron of the P1 transcript. Horizontal lines represent exon-specific probes on the microarray. The signal intensities from the probes in exon 1A or 1B uniquely correspond to the activity of the P1 or P2 promoters, respectively, revealing promoter strength or tissue specificity. Conversely, signals from exon 2 or further downstream exons reflect a mixture of both promoter activities. (B) As annotated in the Known Genes annotations of the UCSC Human Genome Browser (NCBI36), 52% of genes transcribe a single isoform. Of those with multiple isoforms, 60% (7,708) have alternative promoters. (C) the genes with alternative promoters were divided into those with overlap between their first exons or another isoform (57%) and those with mutually exclusive use of their first exon (43%). In total, 3,296 genes contained isoforms with unique first exons. From this set we excluded genes lacking microarray probes or containing repeats in either of the first exons, which would interfere with the hybridization intensity signal. The final analyzed data set contained 1,447 genes.

To identify genes with distinct alternative 5′ exons (Figure 1A), we used the UCSC annotations for human genes (NCBI36) [12], in which one or more start sites are annotated for each gene. In the initial set of 26,304 genes, 12,767 genes had more than one mRNA isoform (Figure 1B; see Methods). Alternative promoters existed in 7,708 genes, of which 57% transcribed a first exon that was not exclusive to the isoform and 42% specifically incorporated a DAlFE into the isoform (Figure 1C). We chose two DAlFE isoforms from every gene, whose first exons do not overlap in their genomic coordinates and do not overlap with any exons of the other isoform. Although genes can have more than two alternative promoters of this type, we found that only 19% of the genes showed that characteristic, indicating that genes with three or more distinct, alternative first exons were relatively uncommon in the genome. To test the gene models underlying our predictions we compared the DAlFEs to experimental CAGE evidence in K562 cells and found that 45% of our set corresponded to CAGE data at the same position, in proximity to TSSs of genes (see Methods).

In all cases, the downstream (P2) promoter was fully embedded in an intron of the P1 transcript (Figure 1A). The median distance between the alternative P1 and P2 transcription start sites (TSS) of the isoforms was 4.8 kb; however, inter-TSS distances fell into two main groups, with median distances of 700 or 30,000 intervening bp (Figure S1A). As first exons of transcripts, DAlFEs were predicted to have no splicing to upstream exons and no participation as alternatively spliced exons of larger transcripts. To test this hypothesis, we scored DAlFEs for the presence of 3′ splice junctions (splice acceptor sites) at their leading edge. Maximum entropy (MaxENT) calculations [13] from DAlFEs were compared to a control set of spliced, internal exons from the same transcripts. The maximum scores of 3′ splice-sites in P1 exons (i.e., upstream DAlFEs) were significantly smaller than those of the internal exons. Median scores were 0.5 versus 9.3 for DAlFEs and internal exons, respectively (*P*-value<2.2×10^−16^, Wilcoxon test) (Figure S1B). Moreover, the distribution of 3′ splice-site scores for P2 exons (i.e., downstream DAlFEs) was virtually identical to that of upstream DAlFEs (Figure S1B).

We postulated that one role of DAlFEs was to contribute regulatory diversity through the role of noncoding secondary structure selectively affecting the rate of translational initiation [7]. Consistent with this idea, 79.1% of DAlFE exons contained only UTR sequences. In contrast, 79.9% of constitutive first exons (CFEs) from single promoter genes had coding sequence in the first exon (a 3.5-fold higher presence; *P*-val<10^−16^, Fisher's exact test). DAlFEs also showed a smaller average size than CFEs by 52 bp (159 vs. 211 bps respectively, *P*-val = 3.3×10^−95^, Student's t-test).

A common regulatory component of promoter sequences is the CpG-island. We examined the DAlFE genes and found that 68% lacked a CpG-island promoter for one of the TSSs. In contrast, CFE genes lacked a CpG-island in only 35% of the promoters (consistent with previous publications) [12]. The large percentage of non-CpG-island promoters in DAlFE genes suggested a different mode of evolution from CpG-island promoters, which could resemble a more ancestral form, such as found in fly, yeast and bacterial promoters, or a more recently acquired, lineage-specific form. The possibility of lineage specific acquisition was addressed through the existence of transposable elements or transposon-derived repetitive elements, which are known to introduce sequences that can be exapted for regulatory roles in the genome [9]; [14]–[16]. To test this hypothesis, we mapped repetitive elements in the first exons and found a 4.3-fold higher occurrence in DAlFEs than CFEs (22.6% and 5.3% respectively; see Methods). To account for the bias of coding sequences against repetitive element insertion, we repeated the analysis using only noncoding CFEs and found that the difference remained significant (2.2-fold higher in DAlFEs, *P*-val = 8.3×10^−22^, Fisher's exact test), indicating that repetitive elements were strongly associated with the presence of alternative first exons. Furthermore, we found that the repetitive elements frequently supplied the TSS used for transcription (2.3-fold higher in DAlFEs, *P*-val = 3.4×10^−16^) (Figure S1C).

Although the presence of repetitive elements within the first exons was informative about alternative promoter evolution, our subsequent analyses dealt with microarray data generated from DAlFE transcripts. We therefore excluded all gene loci that contained a repetitive element in at least one of the first exons (750 out of 3296, 22.8%) to reduce the possibility of artifacts caused by cross hybridization in the assay.

### Global P1 and P2 comparisons

Using the DAlFE dataset, we developed a novel computational method to discriminate the activity of alternative promoters at gene loci by utilizing hybridization probes complementary to the first exons of the transcripts. From the initial set of 3,296 genes whose isoforms used distinct first exons, 1,447 were chosen because they had exon array probes for their first exons that lacked repetitive elements (Figure 1C). We downloaded a public Affymetrix Exon 1.0 array data from 11 human tissues (breast, cerebellum, heart, kidney, liver, muscle, pancreas, prostate, spleen, testes and thyroid), with three biological replicates per tissue, normalized the data using the Affymetrix PLIER software, assessed the median signal of the replicates, and assigned the expression values to the DAlFE probes (see Methods).

### Expression analyses

**Figure 2:**
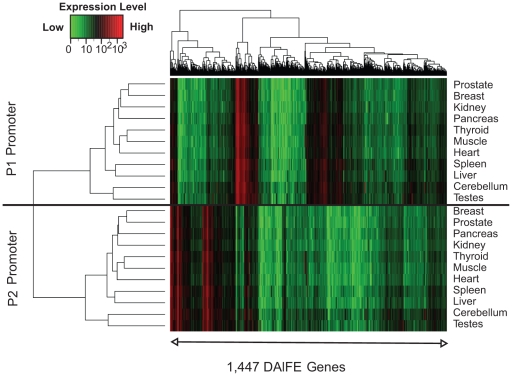
Expression data illustrating the activity of P1 or P2 promoters. Each column represents DAlFE expression from a single gene, with P1 and P2 transcript expression levels depicted in the upper or lower panels, respectively. Expression data represent normalized raw intensities plotted using a red-green scale. Hierarchical clustering was used to order the data from 11 tissues on both the X and Y-axes.

The range of expression data for DAlFE isoforms varied over 3 orders of magnitude from 0 to 4,700 (Figure 2). Comparison of the activity of P1 and P2 promoters across 11 tissues revealed a diversity of patterns, ranging from overlapping expression patterns to mutually exclusive and tissue-specific. Unsupervised hierarchical clustering of the expression intensities over all 11 tissues separated the upstream DAlFEs from their downstream counterparts, indicating that the activities of P1 and P2 promoters were more dissimilar than similar (Figure 2). The median expression intensity for all P2 DAlFEs was slightly lower than the P1 counterparts (22.3 versus 27.1 respectively; see Table 1 for a summary by tissue), but was not significantly different. The distributions of expression intensities conferred by P1 and P2 promoters are similar (Figure S2). All expression values are reported in Table S1.

**Table 1:** Mean and median expression values for each tissue and the Pearson correlation for DAlFE expression levels.

	P1		P2		
Data set	Mean	Median	Mean	Median	Pearson Correlation
All Tissues	104.1	27.1	90.6	22.3	0.03
Breast	76.2	18.9	69.0	16.1	0.03
Cerebellum	142.7	45.3	128.3	44.1	0.03
Heart	86.7	22.5	77.5	17.1	0.00
Kidney	79.1	19.5	67.0	16.1	0.00
Liver	110.4	29.1	92.9	22.6	0.00
Muscle	99.0	23.6	8.52	17.9	0.05
Pancreas	96.1	24.6	81.4	19.7	0.00
Prostate	83.7	20.3	73.9	16.4	0.03
Spleen	131.8	34.7	108.8	26.8	0.13
Testes	126.8	44.4	111.5	39.5	0.04
Thyroid	111.2	28.0	100.7	22.6	0.04

**Figure 3:**
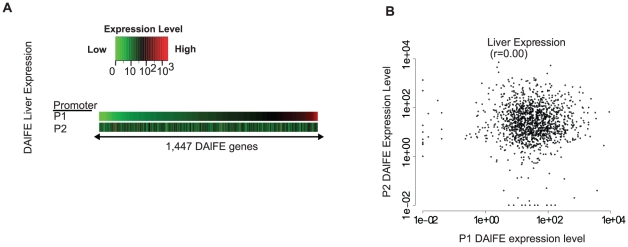
Assessment of coordinated expression patterns among alternative promoters. (A) Pairs of promoters from the same gene are kept together in the same column. Expression data for P1 transcripts in liver are sorted by ascending values. (B) A scatter plot illustrating the independent expression from P1 and P2 promoters in liver. Each point represents the expression level of the P1 promoter on the x-axis and P2 on the y-axis. The Pearson correlation is depicted in the figure (r = 0.00).

Since differential expression levels were identified, we assessed expression intensity values in liver by sorting data relative to the P1 promoter, keeping P1 and P2 pairs together (Figure 3A). In contrast to the ascending values of P1 hybridization signals, P2 transcripts generated a random pattern, indicating no global expression correlation between P1 and P2 promoters. This conclusion held true for the other tissues as well. We also found no correlation among the expression levels of P1 and P2 promoters (Pearson correlation; r = 0.00; Figure 3B). These results verified that the vast majority of alternative promoters were differentially activated. Moreover, the data confirmed that the transcription level of any one isoform should not represent all isoforms from the gene locus.

### Entropy as a quantitative measure for expression uniformity

**Figure 4:**
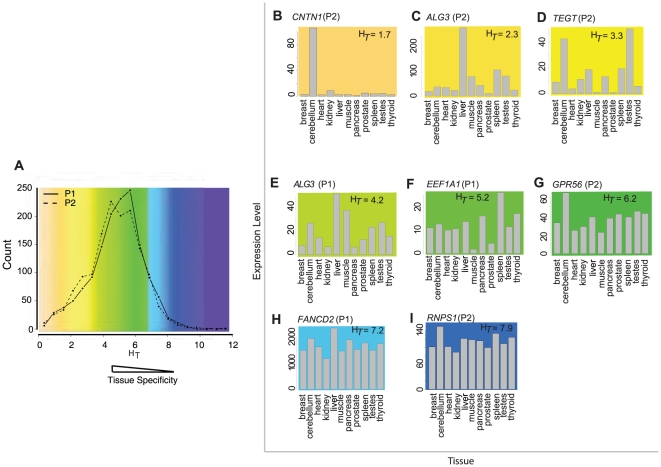
Entropy scores (H_T_) for all isoforms. (A) The histogram plot, overlaid with a color key, shows the distribution of all entropy scores conferred by the P1 or P2 promoters: yellow represents highly tissue-specific, green is moderate, and blue is uniform expression. (B–HI) Expression data from each promoter are shown as bar plots, using he same color key as panel A. Entropy scores are shown in each panel. The y-axis is scaled to each sample.

To objectively quantify tissue-specificity, we used the concept of entropy (H_T_) to measure the deviation in expression uniformity for a given transcript across multiple tissues [17]. For these purposes, ubiquitous expression was defined as the uniformity of expression across tissues regardless of the magnitude of expression whereas tissue-specificity was interpreted as a significant positive deviation from uniformity in one of multiple tissues. Overall, entropy scores ranged from 0.13 (i.e., transcripts expressed predominantly in one tissue) to larger than 10, with a median H_T_ score of 4.9. To gain an impression of the proportion of the data defined as tissue-specific, heterogeneous, or uniformly expressed, a threshold approach was used to bin the transcripts into each category. With H_T_ levels less than 3, tissue-specific expression patterns were found for 14% of the transcripts (Figure 4). Heterogeneous expression occurred for the majority of alternative promoters (79%) (H_T_ values between 3 and 7). Uniform expression identified 7.0% of the transcripts (H_T_ values greater than or equal to 7). Thus 86% of DAlFE transcripts examined in this analysis were expressed in multiple tissues without tissue-specific patterns. All entropy scores are reported in Table S1.

Entropy scores revealed that the position of a promoter within a gene locus (as P1 or P2) did not favor tissue-specific or ubiquitous expression (Figure 4A). Entropy scores also enabled an analysis of the influence of CpG-islands on expression patterns, whose transcripts were hypothesized to have broad rather than tissue-restricted expression patterns. The median entropy scores for CpG-island promoters were higher than non-CpG island promoters (H_T_ = 5.19 versus 4.59; Student's t-test *P*-val = 3.1×10^−35^) (Table S1), indicating that CpG-island promoters had more uniform transcript expression levels. Nevertheless, the CpG-island promoter set was not uniformly active and the non-CpG-island promoter set was not strongly tissue-specific.

The majority of DAlFE transcripts had entropy scores of 3 to 7 (Figure 4A and Table S1). Expression plots for individual genes confirmed the expected patterns by showing increasing uniformity corresponding to increasing H_T_ values (Figure 4B–I). One transcript with a tissue-specific entropy score displayed 100-fold higher expression in cerebellum (i.e., the P2 transcript of *CNTN1*; H_T_ = 1.7) (Figure 4B). The smallest entropy score was 0.13, for the P1 transcript of *PRSS2*, a pancreas-specific trypsinogen enzyme whose expression value in this analysis was 2,805 in pancreas and 0.1 to 7.38 in all other tissues. In contrast, examples of transcripts with uniform expression levels had high entropy scores (H_T_ = 7.88 for P2 of *RNPS1*), which is a part of a surveillance complex against premature stop codons that acts to trigger nonsense-mediated mRNA decay (Figure 4I).

### Assessment of genes with ubiquitous function

**Figure 5:**
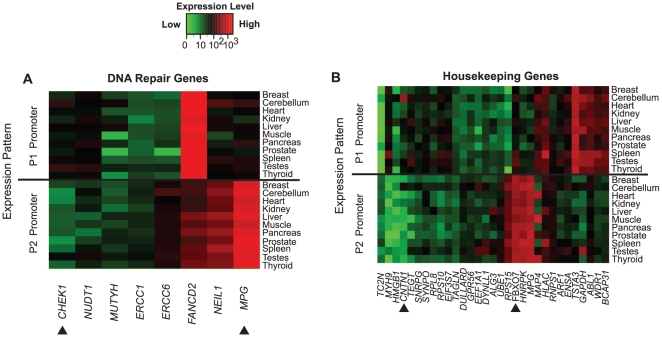
The expression levels and tissue specificities conferred by alternative promoters in a set of DNA-repair genes (A) and a set of housekeeping genes (B) are listed on the x-axes. The upper and lower panels represent P1 and P2 alternative promoters, respectively. Expression levels are indicated by the green to red color scale. Arrowheads indicate the examples from the text.

We performed a case study on a known set of 130 DNA-repair genes [18]. The ubiquitous function of DNA repair genes suggested that single promoters might be sufficient to direct transcription without a need for alternative expression patterns. However, eight genes from the DNA repair set had evidence for a second promoter in our DAlFE annotations. The expression profiles generated by the P1 or P2 promoters of these genes varied across tissues, indicating that DNA repair genes had specialized regulation beyond ubiquitous expression (Figure 5A). For instance, the P1 and P2 isoforms of *CHEK1* (checkpoint homolog, encoding a cell-cycle checkpoint gene) had differential regulation, yet similar entropy scores (H_T_ = 4.35 and 4.39, respectively) and showed the highest expression intensity values in testes (71 or 25, respectively). A literature search of published experimental results revealed northern blot data with multiple bands corroborating high expression of *CHEK1* in testes [19], without examining the contribution of alternative promoters. In contrast to the relatively low level of expression displayed by isoforms of *CHEK1*, isoforms of *NEIL1* and *MPG* showed strong expression for one transcript. A 32-fold difference in expression levels occurred between the P2 and P1 transcripts of *MPG*. *FANCD2* showed strong expression signals for both transcripts.

We subsequently assessed a set of 532 independently characterized housekeeping genes [20] to address the hypothesis that alternative promoters would not contribute significantly to the expression diversity of housekeeping genes. However, 31 genes refuted that expectation by containing alternative promoters with variable expression levels (Figure 5B). For example, *FBXO7* (F-box only protein 7) showed a heterogeneous, moderate-level expression pattern (H_T_ = 3.62) with a seven-fold range from the P1 promoter (47.56 to 371.87) and a high level, ubiquitous expression pattern (H_T_ = 7.0) with 2-fold range from the P2 promoter (612.65 to 1,178.63). Additionally, *CNTN1*, was expressed in all 11 tissues, but showed entropy scores of 1.18 and 1.75 (P1 and P2, respectively). The highest expression was found in cerebellum for P1 and P2 (858.64 and 106.15, respectively), whereas lowest expression was in heart (P1, 14.22) and pancreas (P2, 1.24). Although widely expressed, the increased cerebellar expression of *CNTN1* suggested a more targeted role than a housekeeping function. Consistent with this interpretation, *CNTN1* has a published role as a glycosylphosphatidylinositol (GPI)-anchored neuronal membrane protein that functions as a cell adhesion molecule and exhibits high levels of transcription in brain [21].

### Alternative promoters and tissue preferences

Similarly to the expression data, we analyzed the correlation between entropy scores for P1 and P2 promoters. The Pearson correlation coefficient was 0.15, indicating no overall correlation for tissue-specific activity, although the weakly positive signal suggested that isoforms from some loci might each have tissue-specific expression. To address non-random co-expression of isoforms, we lowered our requirement from tissue-specificity at both promoters in favor of a measurement of “tissue preference” at both promoters, which identified the most favored tissue from a profile allowing heterogeneous expression. The preferred expression levels were identified by a signal that was two-fold higher than the median value for the probe across all tissues (Table S1 and Table 2). Consistent with previously documented expression patterns reported in cerebellum and testes [22], these two tissues held the highest counts of P1 or P2 transcripts with a tissue preference, including 27% of all transcripts expressed principally in cerebellum and 19% in testes (with some overlap). Spleen and liver showed the next highest occurrences of favored expression.

**Table 2:** The breakdown of tissue preferences by count of DAlFE promoters.

	2× Average Expression^a^		Most Preferred^b^		# Expected Pairs^c^	# Observed Pairs^d^	*P*-value for chi-square test
	Count	Percent	Count	Percent			
Breast	26	0.9	15	0.5	0	0	8.44E-01
Cerebellum	795	27.5	677	23.4	79.2	111	2.36E-04
Heart	71	2.5	49	1.7	0.4	2	1.38E-02
Kidney	51	1.8	37	1.3	0.2	4	1.00E-14
Liver	145	5.0	112	3.9	2.2	14	8.71E-16
Muscle	90	3.1	57	2.0	0.6	5	3.11E-09
Pancreas	61	2.1	45	1.6	0.3	4	6.75E-10
Prostate	19	0.7	11	0.4	0	0	8.85E-01
Spleen	239	8.3	189	6.5	6.2	23	1.13E-11
Testes	548	18.9	447	15.4	34.5	73	3.39E-11
Thyroid	77	2.7	32	1.1	0.2	1	5.04E-02
Total			1671	57.7	123.8	237	

a The count (or %) of DAlFE promoters with a higher than expected expression level.

b The count of DAlFE promoters where the tissue was the ‘most preferred’ out of all tissues.

c Expected number for pairs of promoters (P1 and P2) sharing the ‘most preferred tissue’ status in the same tissue.

d Actual number of pairs of promoters sharing the same tissue as the ‘most preferred’.

We examined how often a P1 and P2 promoter pair had the same tissue preference (Table S1). In total, 237 alternative promoter pairs (16%) were predominantly active in the same tissue, almost twice as many as the expected number of 123.8 (see Methods). A chi-square goodness-of-fit test confirmed that this result was highly significant (*P*-val = 2.1×10^−26^) compared to the random expectation. The status of most-preferred tissue was shared for P1 and P2 promoters in cerebellum or testes more than any other tissues. This finding was consistent with abundant transcription in these tissues, but novel regarding the coordinated regulation of alternative promoters in these tissues.

### Tissue preference distinguishes alternative promoter activity

**Figure 6:**
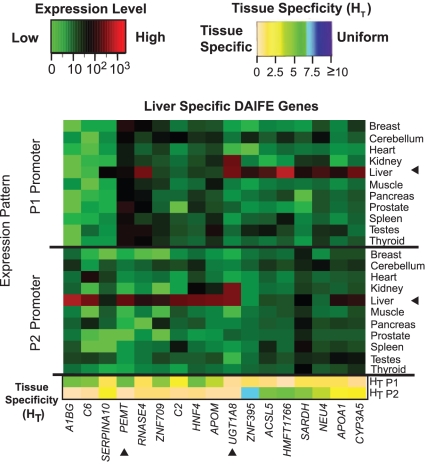
Liver-specific expression attributed to alternative promoters. The heat map illustrates the expression profiles of genes transcribed abundantly in liver compared to other tissues. Peak expression values (red cells) corresponding to liver-specific expression were produced from either the P1 or P2 promoters. The tissue specificity scores (H_T_) are shown in the lower two rows. Arrowheads denote liver from other tissues and indicate examples described in the text.

We found that most often only one promoter of a gene showed a tissue preference. For example, P1 of *PEMT* (phosphatidylethanolamine N-methyltransferase) had an entropy score of 5.7 versus 0.6 for P2 (liver-specific) (Figure 6). These data were consistent with previously published work that identified P2 of *PEMT* as liver-specific [23]. Additional isoforms with liver-specific expression were equivalently divided between P1 and P2 activity. One third of those liver specific genes showed co-expression of the P1 and P2 promoters in a liver-specific manner (Figure 6). Only one gene, *UGT1A8*, had dual promoter expression that was specific to liver and kidney, which contradicts the published literature reporting only extra-hepatic expression, but is consistent with the recent publication of Li et al. [24], which documents expression in the liver.

To assess whether the expression patterns had biological relevance, a gene ontology analysis (using GOSTAT) was performed on all genes showing a preference for liver expression from either promoter. The results confirmed that the functional roles of the genes were consistent with liver-related functions, albeit from several types of liver cells. For example, the GO terms for carboxylic, organic acid and lipid metabolic processes were likely contributed by genes that were active in liver mitochondria, which heavily populate hepatic cells (Table 3). In contrast, the term humoral immune response was likely contributed by resident Kupffer cells, which are specialized macrophages located in the liver [25].

**Table 3:** Best GO terms for molecular function in the liver-preferred dataset.

Best GOs	GO Accession	Genes	Count	Total	*P*-Value
			(67)	(33, 972)	
Carboxylic metabolic process	GO:0019752	*ACSL5*, *SHMT2*, *SCYE1*, *SARDH*, *LTB4DH*, *AHCY*, *GLUD1*, *IDH1*, *CHGN*, *NR1H4*, *HSD17B4*	11	795	0.000164
Organic acid metabolic process	GO:0006082	*ACSL5*, *SHMT2*, *SCYE1*, *SARDH*, *LTB4DH*, *AHCY*, *GLUD1*, *IDH1*, *CHGN*, *NR1H4*, *HSD17B4*	11	798	0.000164
Humoral immune response	GO:0006959	*C4BPB*, *LTF*, *C2*, *C6*	4	75	0.00249
Lipid metabolic process	GO:0006629	*ACSL5*, *LTB4DH*, *CYP3A5*, *HNF4A*, *PEMT*, *APOA1*, *APOM*, *NR1I2*, *NR1H4*, *HSD17B4*	10	946	0.00249
Cellular lipid metabolic process	GO:0044255	*ACSL5*, *LTB4DH*, *CYP3A5*, *PEMT*, *APOA1*, *APOM*, *NR1I2*, *HSD17B4*, *NR1H4*	9	768	0.00249
Monocarboxylic acid metabolic process	GO:0032787	*ACSL5*, *IDH1*, *LTB4DH*, *CHGN*, *NR1H4*, *HSD17B4*	6	287	0.00249
Lipid transporter activity	GO:0005319	*PCTP*, *APOA1*, *APOM*, *HSD17B4*	4	96	0.00249
Steroid hormone receptor activity	GO:0003707	*NR1H3*, *HNF4A*, *NR1I2*, *NR1H4*	4	117	0.004

**Figure 7:**
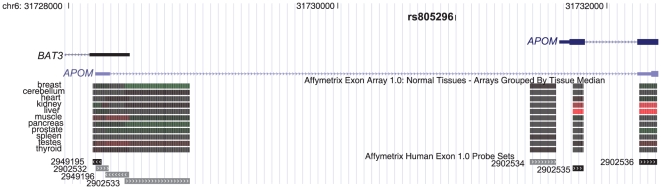
P1 and P2 of *APOM*. The type 2 diabetes-associated SNP rs805926 is shown as a custom track in the UCSC Human Genome Browser. Two transcripts of the *APOM* gene are oriented from left to right, whereas the transcript for *BAT3* is oriented from right to left. Exon array data from Affymetrix for 11 tissues is shown below the gene models. Red bars indicate high expression and green bars correspond to low level expression. Individual probes for the exon-microarray are shown in the bottom panel. The SNP corresponds to the P2 promoter region of *APOM*, which shows strong expression in liver.

Additional examples of a specific expression patterns conferred predominantly from one promoter include *PDZD4* (P1; cerebellum), *C6* (P2; liver), *ASB5* (P1; muscle), *PPP1R12B* (P2; heart), *SLC5A12* (P2; kidney), *APOM* (P2; liver), *ADPRHL1* (P1; heart), and *TP73L* (P1; muscle) (Table S1). Our data matched published literature for the expression patterns attributed to these gene loci [26]–[33]; however, the contribution of alternative promoters was rarely addressed in those studies. The relevance of alternative promoters in mechanisms of disease is apparent in the tissue-preferences identified for these isoforms. For example, the *PDZD4* gene was reported to be upregulated in 13 of 13 synovial sarcomas (SS) [26], whereas siRNA against *PDZD4* resulted in suppression of SS tumor cell growth. Alternative splicing of *PDZD4* coding exons was reported in Nagayama et al. [26], but the presence of an alternative promoter was not. *APOM*, known for cholesterol regulation [31], confers risk for the development of type 2 diabetes through SNP T-778C in the proximal promoter region among Han Chinese [34]. This SNP (rs805295) maps to a region of the P2 promoter, consistent with our data showing that P2 has liver-specific expression (H_T_ = 0.8; 626.49 expression value) (Figure 7). *TP73L*, known for monogenic malformation syndromes such as cleft palate [33] (suggesting a tissue-specific role), has strong sequence similarity to the tumor suppressor *TP53*. Its isoforms, which are created by alternative promoters and alternative splicing, have divergent abilities to transactivate p53 reporter genes and induce apoptosis [33] (suggesting a more generalized role). The P1 transcript showed strongest expression in muscle (H_T_ = 1.2), whereas P2 showed heterogeneous expression patterns (H_T_ = 5.66). *C6*, traditionally known as a liver-specific gene [27], showed preferential P2 activity in liver (H_T_ = 0.87; expression value 636.36) as well as in heart (expression value 141.21) (Table S1). These results are consistent with published data stating that the human heart expresses all mRNAs and proteins for the classical complement pathway; furthermore that expression of these genes becomes upregulated in areas of myocardial infarct, causing myocardial damage that may become chronic [35]. The expression level of transcript P1 was 0.54 in heart, strongly implicating the P2 transcript in disease processes of the heart.

### Case Study – *HNF4A*

Mutations in the *HNF4A* gene are documented dominantly inherited maturity onset diabetes of the young (MODY) [36]. Two *HNF4A* promoters have been identified, one with kidney and liver expression (downstream, but called P1 in the literature) and one with pancreatic-specific expression (upstream, but called P2 in the literature) (Figure S3). Thus far, three MODY-associated mutations have been described in the P2 promoter coinciding with transcription factor binding sites for IPF1, HNF1a, or HNF1a/b [36]. Data generated in this analysis confirmed the preferred liver and kidney expression of the downstream promoter (H_T_ = 1.25) (expression levels of 540.15 in liver and 92.45 in kidney). In contrast, the upstream promoter had heterogeneous expression (H_T_ = 5.63) with its highest expression in pancreas (signal intensity 32.15), prostate (signal intensity 39.7) and cerebellum (signal intensity 36.36) (Table S1 and Table S2). Consistent with previous reports, pancreatic expression levels of the upstream transcript were higher than the downstream transcript [37], [38] and liver expression levels were higher for the downstream transcript than the upstream transcript [39]. As with our results, low-level expression has been documented for tissues besides liver and kidney from the downstream promoter [38], [40].

### Comparison to public repositories

Comparison of the alternative transcript annotations and expression data to the cDNA microarray data from the Genomics Institute of the Novartis Research Foundation (GNF) [41], showed that the majority of DAlFE isoforms were not assessed in the GNF expression platform. Specifically, GNF expression data quantified expression from both of our isoforms in 313 out of the 1,447 genes examined (21%), whereas the rest had a single expression profile assigned to their locus. Assessment of our collection of transcripts in the UNIPROT database [42] indicated that only 18% of all transcripts (534 of 2,894 isoforms) had a citation for tissue-specific expression, whereas 57% (1,671 of 2,894) showed a preference for a tissue in our analysis, illustrating that distinct expression profiles of isoforms are far more complex than indicated in most repositories.

### Epigenetic analyses

**Figure 8:**
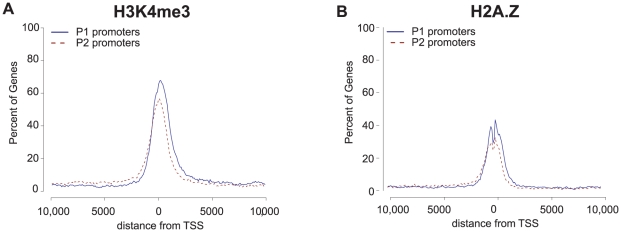
Characterization of the histone modification domains for P1 and P2 alternative promoters (in CD4+ T Cells). (A) ChIP-Seq data for the histone modification H3K4me3 and (B) the histone H2A.Z plotted against the percent of genes containing the tags. The position of the ChIP-Seq tags was plotted as the distance relative to the transcription start sites (TSSs) in base pairs. A minimum density of 3 sequence tags overlapping a 100 bp window was required for inclusion.

The independence of the P1 and P2 promoters suggests that regulatory characteristics should appear independently at each site. We examined epigenetic data associated with the activation of promoters. Specifically, the tri-methylation of the fourth lysine on histone H3 (H3K4me3) is known as a mark of gene activation associated with promoters [43]. Using available chromatin-immunoprecipitation sequencing (ChIP-seq) data (for CD4+ T-cells) [43], we found a significant enrichment of H3K4me3 around the TSSs of P1 and P2 promoters (Figure 8A), with similar peak heights. Similarly [43], the H2A.Z histone variant (Figure 8B), which is associated with active transcription in mammalian sequences [44] had similar peak heights for P1 and P2 promoters, although lower than H3K4me3.

**Figure 9:**
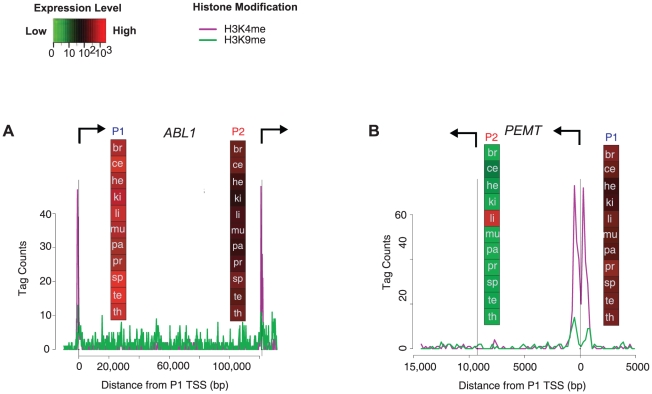
Histone modifications in alternative promoters. The P1 and P2 promoters of *ABL1* (A) and *PEMT* (B) are shown with arrows at the transcription start sites indicating direction of transcription. The H3K4me3 and H3K9me1 histone modifications are from CD4+ T-cells and correspond to the activation of promoters in that cell type. The vertical panels represent expression intensity signals as color-coded boxes, with abbreviations for the 11 tissues used in this study (brain, cerebellum, heart, kidney, liver, muscle, pancreas, prostate, spleen, testes, thyroid). The P2 form of *PEMT* did not show the H3K4me3 activation signal in CD4+ T-cells, as expected, since its expression was specific to liver. Each promoter had its own domain of histone modifications, whereas intervening regions did not show enrichment.

We plotted tag counts of H3K4me3 and H3K9me1 at *ABL1* (Abelson murine leukemia viral oncogene) and *PEMT* as examples. *ABL1* was chosen since it was ubiquitously expressed from both promoters at high levels across the 11 tissues, and was likely to show a similar trend in CD4+ T-cells. This hypothesis was confirmed by the histone methylation data from CD4+ cells (Figure 9A). In contrast, our analysis showed that *PEMT* was only ubiquitously expressed from the P1 promoter, whereas the P2 promoter activity was restricted to the liver. Consistent with this data, the P1 promoter of *PEMT* showed the activation marks of histone methylation in CD4+ T-cells, whereas the P2 promoter did not (Figure 9B). These results illustrate the presence of distinct epigenetic marks at active and inactive promoters of a gene. Overall, these methylation marks indicate that active, P1 or P2 promoters could be identified by their epigenetic features, regardless of the status of other promoters in the locus. The signals were identical at the P1 and P2 promoters when transcription was active at both sites, whereas the signals were opposite when only one promoter was active. This analysis also helped in confirming that gene annotations correctly identified promoter regions of *ABL1* and *PEMT* by their distinct chromatin features [44].

**Figure 10:**
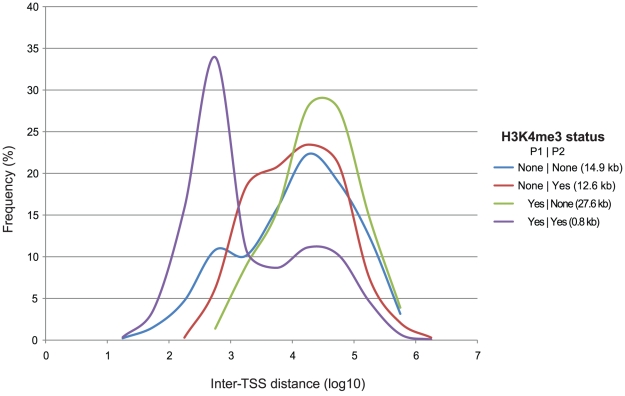
Frequency of H3K4me3 modification versus inter-TSS distance. DAlFE genes were annotated for their H3K4me3 status (NONE or YES) for P1 and P2 promoters and plotted against the corresponding inter-TSS distances. Modification status is labeled in the order P1|P2. The median inter-TSS distance for each category is listed in parentheses.

Extending the analysis of chromatin marks to the full set of DAlFE genes showed that 34.4% have H3K4me3 at one or the other promoter, but not both, as indicated by their overlap with “tag islands” defined for this histone modification (see Methods). The modifications also identified 52% of genes that share signal for H3K4me3 at P1 and P2, and 13% that have no marks at either promoter in CD4+ T-cells. The median distance between alternative promoters was relatively short for genes with simultaneous H3K4me3 modifications (800 bp) (Figure 10), suggesting that promoters with coordinated modifications or those coordinately lacking modifications should share this inter-TSS characteristic. However, we found that promoters coordinately lacking the modifications had the opposite profile, in which the median inter-TSS distance was 14.9 kb. Median distances greater than 12 kb were measured for those sets of promoters with mutually exclusive modifications as well. Combined with other data in this analysis, we concluded that a slight majority of alternative promoters contain epigenetic marks indicative of concurrent activation, likely due to their close spatial proximity. However, the absence of strong correlations for transcriptional output levels or tissue-preferences indicates that alternative promoters confer targeted and specialized functions to their transcripts and do not represent a mechanism for redundant activity.

## Discussion

Alternative promoters are recognized for creating transcript diversity in a cell, with mostly uncharacterized consequences. Of the different types of alternative promoters, our analysis focused on the subset that utilized distinct first exons. In a novel method to address expression diversity in the cell, we analyzed microarray data to measure transcription levels and tissue preferences. Consistent with the conclusion of Faulkner and Carninci, 2009 [16] we found that repetitive elements are associated with alternative 5′ ends and upstream regions of some transcripts. These data indicate that sequences of repetitive elements contribute to the function of alternative promoters and imply that repetitive elements selectively contribute to the formation of lineage-specific alternative start sites.

In this analysis the expression patterns of distinct first exons served as a proxy for the activity of the promoter and all associated distal regulatory elements. Although the analysis utilized existing annotations of 5′ ends of transcripts (i.e. in UCSC Known Genes), the interpretation of promoter activity was associated directly with the expression data and was not dependent on the precise endpoints of the exon annotations. Therefore, each distinct first exon corresponded to an expression profile that was uniquely its own, revealing the specificity and transcriptional activation of the associated promoter. Individual regulatory signals contributed by distal elements were not evaluated in this analysis. Consistent with a role in transcriptome diversification, the expression levels and tissue preferences of promoters P1 and P2 in each gene were markedly different. The diversity indicated that the conventional descriptions of genes, which routinely include only a single transcript or a mixture of all transcripts, omit important details about the biological functions contributed by individual isoforms. Supporting this conclusion, genes classified as ubiquitous or tissue-specific were found to contain alternative isoforms with different expression profiles. The presence of such examples indicates that genes can undergo sub-functionalization through the appearance of distinct isoforms, comparable to sub-functionalization that occurs through the creation of paralogous genes. Our results also show that alternative promoters maintain their independent expression patterns through the presence of distinct epigenetic marks. These data confirm that the regulation of alternative promoters is a targeted rather than passive feature. Recent methylation analyses [45] corroborate this conclusion by showing that intragenic CpG-islands (putative promoters) are often silenced compared to 5′ CpG-island promoters (in brain), indicating that downstream promoters remain inactive despite the presence of active transcription throughout the locus. Moreover, different cellular environments activate alternative promoters to different extents. For example, we found that cerebellum has more transcripts expressed preferentially and coordinately than other tissues. This result suggests a more permissive environment in cerebellum, however we recognize the fact that cerebellum is a complex tissue that contains a collection of many heterogeneous cell types whose unique transcription patterns could be mixed together.

Altogether this analysis indicates that the further characterization of alternative promoter activity will help elucidate the biological complexities of every cell type. Addtional studies will also extend the functional classification of genomic DNA sequences, which remain largely under-characterized. As sequence polymorphisms are likely to be identified in these functional regions, they represent candidates for altering gene expression and causing complex diseases [46]. Noncoding SNPs affecting gene expression are being identified in genome-wide association testing [47] and experimental validation of the role of these sequences is a necessity for biological interpretation. Further characterization of alternative promoters and their products will extend to discovery of co-regulatory mechanisms of gene expression, since related transcription factor binding sites should control expression in the same tissues. Ultimately, transcript-isoform information and the use of alternative promoters should be considered in all genomic analyses. A small number of databases currently provide information on alternative transcripts, such as FANTOM4 EdgeExpressDB [48]. As independently regulated mRNA isoforms gain more appreciation, the definition of a gene [49] may expand to address different cellular functions for each isoform.

Despite the emphasis on promoters as major regulatory elements of transcription, the act of generating mRNA in a cell is a complex problem. Promoters work collectively with enhancers to confer cell-type appropriate levels of transcription. Moreover, microRNA can selectively down-regulate the accumulation of transcripts in a cell or interfere with the process of translation. Without an integrated picture of the regulatory components of a cell, the basic information necessary for understanding regulatory networks and therapeutic interventions to disease may remain incomplete.

## Methods

We used the definition of a gene as a set of transcripts that share sequences [49], which corresponded to the “clusterId” field in the “knownIsoforms” Table of the UCSC Genome Browser (hg18), where each cluster represents a gene (http://genome-test.cse.ucsc.edu/goldenPath/gbdDescriptions.html# KnownIsoforms). This analysis was run on the human genome assembly hg18. The gene clusters represent summary-level annotations integrating information from all transcripts in the same genomic locus. Our pipeline records this information as the total number of clusters. Custom software was developed for these analyses.

### Identification of DAlFE Gene Set

We identified all isoform clusters for which there are at least 2 transcripts with a unique first exon. Given this definition, we were able to use the strand and exon position columns from the “KnownGene” Table of the Genome Browser for human genome assembly 18 to identify genes whose 5′ exon of an alternative downstream (or P2) transcript was completely contained within an intron of an upstream (or P1) transcript, as shown in Figure 1. Only the first two promoters encountered along the DNA sequence were considered for any given gene. All genes that contain non-standard exonic features were removed. This includes first exons that had repetitive elements (via REPEATMASKER), or those that were too short for adequate detection in a microarray analysis (60 bp or less). To examine the occurrence of coding sequence in DAlFEs, we constructed a control set of 9,929 CFEs from loci with at least three exons and a first exon that is shared by all isoforms. MaxENT splice-site scores were generated using software from the Burge lab at MIT, http://genes.mit.edu/burgelab/maxent/download/fordownload.tar.gz.

### Assignment of probe accession numbers

Once filtered, the DAlFE dataset was compared to the exons represented on the Affymetrix exon array. This step was done through a table join at Galaxy2 database (www.bx.psu.edu). The join function returned probe accession numbers for each exon. These numbers served as the input for the Affymetrix software, ExACT, which provided access to microarray data collected from 3 replicates each of 11 tissues (including breast, cerebellum, heart, kidney, liver, muscle, pancreas, prostate, spleen, testes and thyroid).

### Expression Data

The expression data were generated from Human Exon 1.0 ST microarrays by Affymetrix (Santa Clara, CA). The data is publicly available from Affymetrix (http://www.affymetrix.com/support/technical/sample_data/exon_array_data.affx), in GeneChip Operating Software flat file format. The GEO platform platform ID is GPL5175. Each microarray contained 5 million probes, with approximately four probes per exon. Probes were at least 25 bp long with a median length of 123 bp. Approximately 1000 anti-genomic probes were used for background subtraction, categorized by GC count. In the expression data, all exons had three intensity values corresponding to their expression levels in triplicate assays for 11 human tissues: breast, cerebellum, heart, kidney, liver, muscle, pancreas, prostate, spleen, testes and thyroid. Data were analyzed with the Affymetrix Expression Console tool using the Probe Logarithmic Error Intensity Estimate summary method and sketch quantile normalization (http://www.affymetrix.com/analysis/index.affx). The normalized expression values varied from an intensity of near 0 to just above 10,000, with median values of 27.1 and 22.3 from the P1 and P2 promoters, respectively.

Median expression values for the three intensity values were calculated, disregarding values exceeding 3.5-fold difference from the median. Using this criterion 5.6% of the probe values were excluded by being too far above and 2.8% too far below the median. This method created a continuum of probe values, for which only neighboring categories showed overlap (Figure S4). Therefore, when comparing probe values we only interpreted changes >2-fold to be significantly different. Consequently, in almost all comparisons the median probe values differ by orders of magnitude and derive from raw probe values with no overlap.

These data were consistent with results produced by Affymetrix (Figure S5), despite slightly different normalization procedures (within tissues, versus across all tissues). The Affymetrix data had a maximum intensity of just over 10,000 as well, but the minimum value was two, rather than zero. The median values in the Affymetrix data were 29.1 and 23.8 for the P1 and P2 promoters, respectively.

### CAGE data

The following UCSC Genome Browser datasets were used for “− strand” transcripts:

http://hgdownload.cse.ucsc.edu/goldenPath/hg18/database/wgEncodeRikenCageMinusClustersK562NucleusLongpolya.txt.gz or for “+ strand” transcripts:

http://hgdownload.cse.ucsc.edu/goldenPath/hg18/database/wgEncodeRikenCagePlusClustersK562NucleusLongpolya.txt.gz. The 3292 tags on the “+ strand” and 3300 on the “− strand” were examined for overlap with the DAlFE isoforms within distances of 100 bps (using a 201-nt interval centered on the TSS) or within 50 bp (using a 101-nt interval centered on the TSS).

### Repetitive Elements

Repetitive elements were obtained from the UCSC Genome Browser hg18 RepeatMasker annotation track. We excluded repeats from “Simple_repeat”, “Satellite” and “Low_complexity” categories.

### Entropy

As a measure of tissue-specificity, we applied Shannon entropy (H), similar to Schug et al. [17]. We used a transformed version (H_T_) to normalize the distribution of values. H_T_ is shown in Equation 1, where T is the sum of all expression values, e_t_ is the expression in tissue T, and n is the number of tissues (11, for our data set).
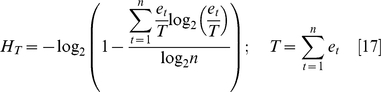
We added a small arbitrary number (5) to each intensity value (e_t_) before calculating entropy in order to avoid misleading readings for low expression values. Otherwise, an intensity value of 5 compared to a value of 0.1 would look like a 50-fold change, even though noise alone could account for the difference.

### Shared tissue preferences

To calculate the expected value, if 23.4% of the promoters showed a preference for cerebellum, the expectation is that 5.5% (or 0.234^2^) of the promoter pairs should have a preference for activity in cerebellum (0.234^2^×1447 = 79.2). These values are shown in the Expected Pairs column of Table 2. However, we observed 111 promoter pairs with a preference for cerebellum (see the Observed Pairs column). Overall, 123.8 promoter pairs were expected to share a tissue preference, while almost twice as many (237) were observed. A chi-square goodness-of-fit test confirmed that this result was highly significant (p = 2.1×10^−26^).

### Histone Modifications

Tag islands for H4K4me3 histone modifications were computed following the Barski et al. [43] algorithm. Each of the P1 and P2 TSSs for 3,296 genes were overlapped with the tag islands to determine their inclusion status.

## Supporting Information

Figure S1 (A) The distribution of the distances between transcription-start sites of pairs of upstream and downstream promoters. The full set of DAlFEs containing 3,296 genes and the restricted set used in the expression analyses (1,447 genes) are plotted with solid and dashed lines, respectively. (B) MaxENT scores of 3′ splice-sites at DAlFEs or second exons in the same transcript. (C) The profile of repetitive element occurrence (%) in a 300-bp window centered on the TSS of DAlFE and CFE transcripts. For comparison we used only non-coding exons including 3,001 DAlEs (blue) and 1,200 CFEs (red).
(0.07 MB PDF)

Figure S2 The log10 expression data for transcripts in all tissues, grouped as P1 or P2 transcript isoforms.
(0.13 MB PDF)

Figure S3 (A) Illustration of the *HNF4A* locus with alternative promoters P2 and P1. Terminal exons from the P1 promoter are shaded white, whereas the 5-prime P2 exon has a dashed border. Exons shared by both forms are gray with solid borders. (B) In the absence of alternative splicing, the coding sequences generated from the P2 and P1 promoters differ at their amino termini.
(0.95 MB PDF)

Figure S4 Measurement of variation among replicate probe intensities. The variation of raw probe intensities including three replicates for every probe is shown on the y-axis. The range of mean values is shown on the x-axis. Each vertical bar represents probe values whose mean was near the x value; for example, the bar at 10 represents values between 8.91 (100.95) and 11.22 (101.05). Each box represents 50% of the data points and all remaining points are within the dotted lines. The data illustrate that signal intensities varying by orders of magnitude represented significantly different values, i.e. the mean probe intensities at 10 do not overlap those at 100 or 1000.
(0.16 MB PDF)

Figure S5 A scatter plot of the log10 expression values calculated for DAlFE exons compared to the data set analyzed by Affymetrix (available on the UCSC Genome Browser), showing a strong correlation. The Affymetrix set had a minimum value of two, whereas the DAlFE expression values started at zero.
(0.19 MB PDF)

Table S1 All expression values and entropy scores.
(0.42 MB XLSX)

Table S2 *HNF4* data.
(0.05 MB DOC)
