# Supplementary figures and images for: Tissue-Specific and Ubiquitous Expression Patterns from Alternative Promoters of Human Genes
Source: PLoS One. 2010 Aug 18;5(8):e12274. doi: 10.1371/journal.pone.0012274 (PMC2923625; doi:10.1371/journal.pone.0012274)

A

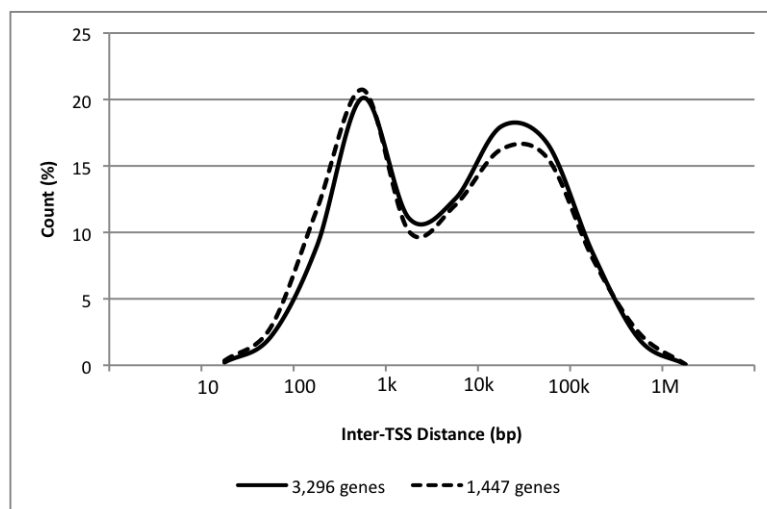

B

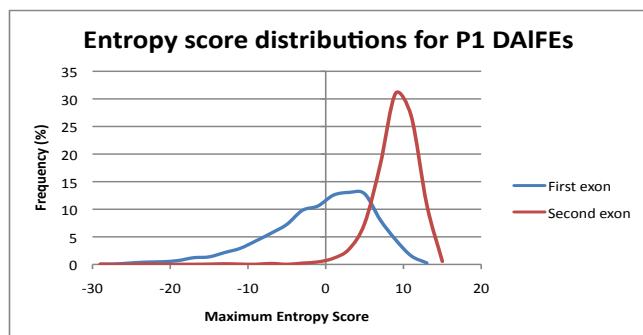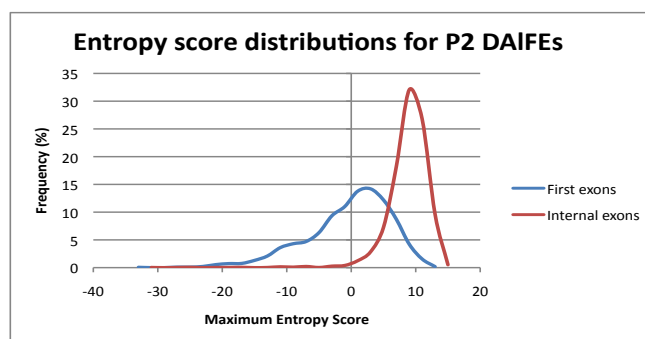

C

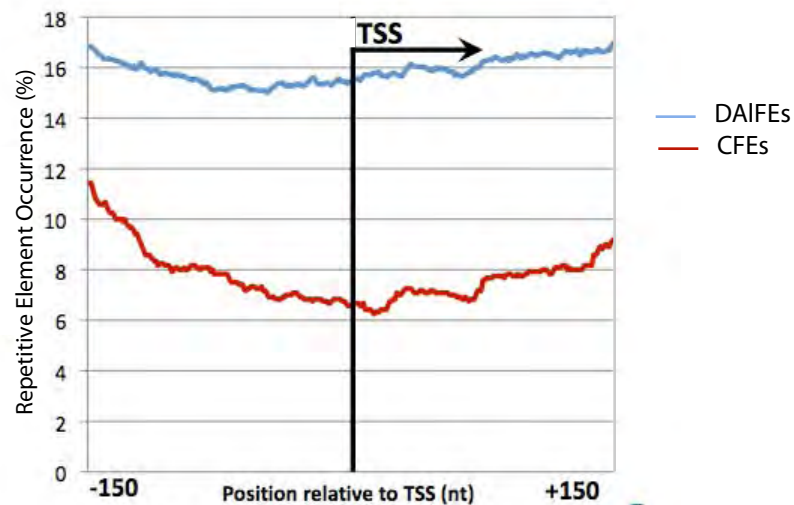

Supplement: Figure S1 — (A) The distribution of the distances between transcription-start sites of pairs of upstream and downstream promoters. The full set of DAlFEs containing 3,296 genes and the restricted set used in the expression analyses (1,447 genes) are plotted with solid and dashed lines, respectively. (B) MaxENT scores of 3′ splice-sites at DAlFEs or second exons in the same transcript. (C) The profile of repetitive element occurrence (%) in a 300-bp window centered on the TSS of DAlFE and CFE transcripts. For comparison we used only non-coding exons including 3,001 DAlEs (blue) and 1,200 CFEs (red). (0.07 MB PDF) [file pone.0012274.s001.pdf]

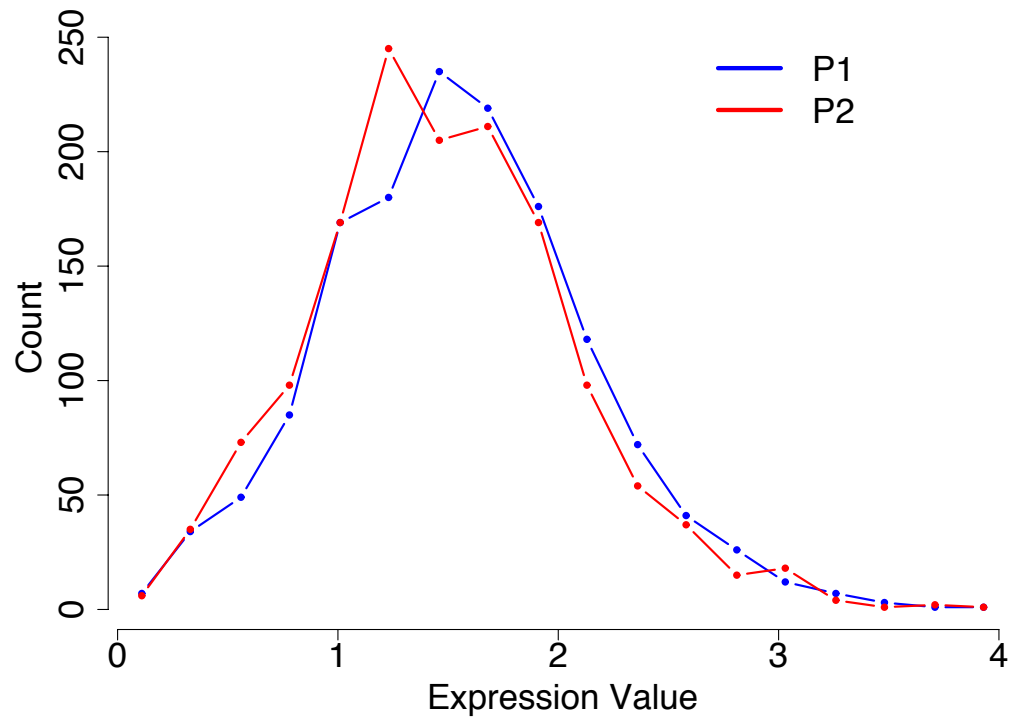

Supplement: Figure S2 — The log10 expression data for transcripts in all tissues, grouped as P1 or P2 transcript isoforms. (0.13 MB PDF) [file pone.0012274.s002.pdf]

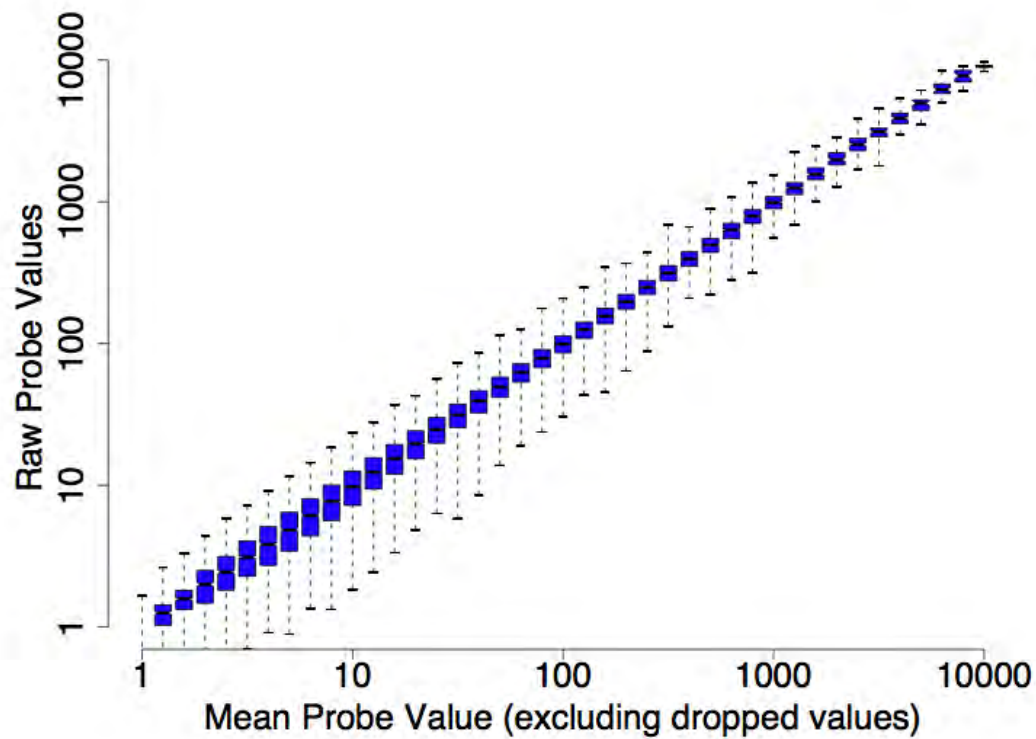

Supplement: Figure S4 — Measurement of variation among replicate probe intensities. The variation of raw probe intensities including three replicates for every probe is shown on the y-axis. The range of mean values is shown on the x-axis. Each vertical bar represents probe values whose mean was near the x value; for example, the bar at 10 represents values between 8.91 (100.95) and 11.22 (101.05). Each box represents 50% of the data points and all remaining points are within the dotted lines. The data illustrate that signal intensities varying by orders of magnitude represented significantly different values, i.e. the mean probe intensities at 10 do not overlap those at 100 or 1000. (0.16 MB PDF) [file pone.0012274.s004.pdf]

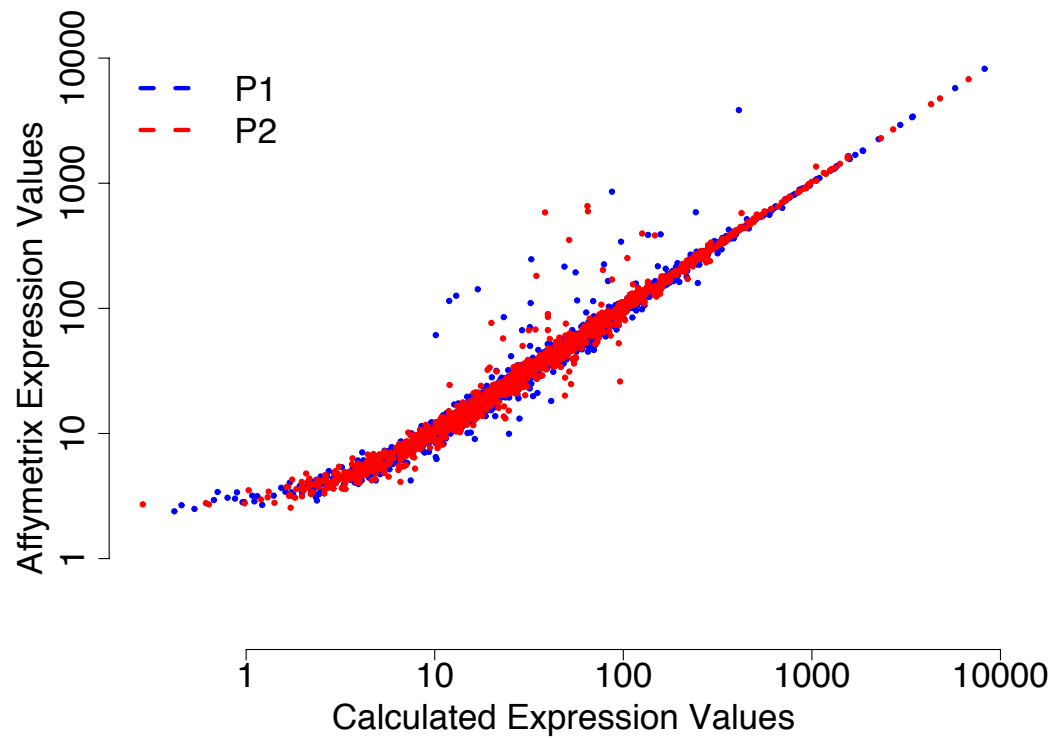

Supplement: Figure S5 — A scatter plot of the log10 expression values calculated for DAlFE exons compared to the data set analyzed by Affymetrix (available on the UCSC Genome Browser), showing a strong correlation. The Affymetrix set had a minimum value of two, whereas the DAlFE expression values started at zero. (0.19 MB PDF) [file pone.0012274.s005.pdf]
