# Supplementary material for: Tissue-Specific and Ubiquitous Expression Patterns from Alternative Promoters of Human Genes
Source: PLoS One. 2010 Aug 18;5(8):e12274. doi: 10.1371/journal.pone.0012274 (PMC2923625; doi:10.1371/journal.pone.0012274)
Supplement: Figure S3 — (A) Illustration of the HNF4A locus with alternative promoters P2 and P1. Terminal exons from the P1 promoter are shaded white, whereas the 5-prime P2 exon has a dashed border. Exons shared by both forms are gray with solid borders. (B) In the absence of alternative splicing, the coding sequences generated from the P2 and P1 promoters differ at their amino termini. (0.95 MB PDF) [file pone.0012274.s003.pdf]

A

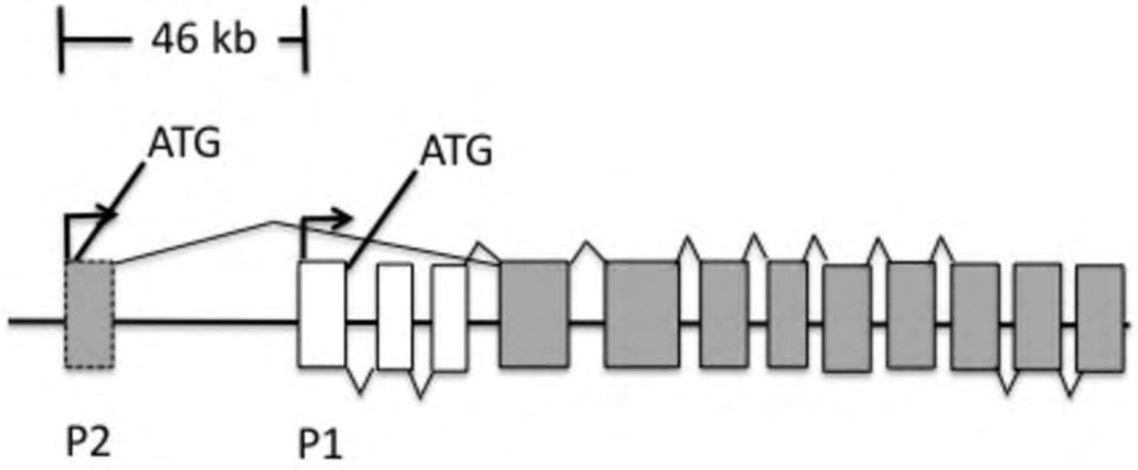

B

```
CLUSTAL 2.0.12 multiple sequence alignment

Downstream      MILLPLRLARLRHPLRHHWSISGGVDSSPQGDTSPSEGTNLNAPNSLGVSALCAICGDRA 60
Upstream        -------MVSVNAPLG------APVESS--YDTSPSEGTNLNAPNSLGVSALCAICGDRA 45
                       :. :. **       . *:**   *****************************

Downstream      TGKHYGASSCDGCKGFFRRSVRKNHMYSCRFSRQCVVDKDKRNQCRYCRLKKCFRAGMKK 120
Upstream        TGKHYGASSCDGCKGFFRRSVRKNHMYSCRFSRQCVVDKDKRNQCRYCRLKKCFRAGMKK 105
                ************************************************************

Downstream      EAVQNERDRISTRRSSYEDSSLPSINALLQAEVLSRQITSPVSGINGDIRAKKIASIADV 180
Upstream        EAVQNERDRISTRRSSYEDSSLPSINALLQAEVLSRQITSPVSGINGDIRAKKIASIADV 165
                ************************************************************

Downstream      CESMKEQLLVLVEWAKYIPAFCELPLDDQVALLRAHAGEHLLLGATKRSMVFKDVLLLGN 240
Upstream        CESMKEQLLVLVEWAKYIPAFCELPLDDQVALLRAHAGEHLLLGATKRSMVFKDVLLLGN 225
                ************************************************************

Downstream      DYIVPRHCPELAEMSRVSIRILDELVLPFQELQIDDNEYAYLKAIIFFDPDAKGLSDPGK 300
Upstream        DYIVPRHCPELAEMSRVSIRILDELVLPFQELQIDDNEYAYLKAIIFFDPDAKGLSDPGK 285
                ************************************************************

Downstream      IKRLRSQVQVSLEDYINDRQYDSRGRFGELLLLLPTLQSITWQMIEQIQFIKLFGMAKID 360
Upstream        IKRLRSQVQVSLEDYINDRQYDSRGRFGELLLLLPTLQSITWQMIEQIQFIKLFGMAKID 345
                ************************************************************

Downstream      NLLQEMLLGGSPSDAPHAHHPLHPHLMQEHMGTNVIVANTMPTHLSNGQMCEWPRPRGQA 420
Upstream        NLLQEMLLGGSPSDAPHAHHPLHPHLMQEHMGTNVIVANTMPTHLSNGQMCEWPRPRGQA 405
                ************************************************************

Downstream      ATPETPQPSPPGGSGSEPYKLLPGAVATIVKPLSAIPQPTITKQEVI 467
Upstream        ATPETPQPSPPGGSGSEPYKLLPGAVATIVKPLSAIPQPTITKQEVI 452
                ***********************************************
```
