# Supplementary material for: Tissue-Specific and Ubiquitous Expression Patterns from Alternative Promoters of Human Genes
Source: PLoS One. 2010 Aug 18;5(8):e12274. doi: 10.1371/journal.pone.0012274 (PMC2923625; doi:10.1371/journal.pone.0012274)
Supplement: Table S2 — HNF4 data. (0.05 MB DOC) [file pone.0012274.s007.doc]

**Table S2.** P1 and P2 transcript expression levels of *HNFA*

| Features | Promoter | Upstream (P2) | Downstream (P1) |
| --- | --- | --- | --- |
|  | Gene name | *HNF4A* | *HNF4A* |
|  | CGI status | Non-CGI | Non-CGI |
|  | TSS distance | 45,483 | N/A |
|  | Probe ID | uc002xlu.1 | uc002xma.1 |
| Expression level | Breast | 22.7 | 11.7 |
|  | Cerebellum | 36.3 | 44.8 |
|  | Heart | 17.0 | 26.0 |
|  | Kidney | 22.0 | **92.4** |
|  | Liver | 27.0 | **540.1** |
|  | Muscle | 12.6 | 10.5 |
|  | Pancreas | **32.1** | 20.4 |
|  | Prostate | 39.7 | 5.0 |
|  | Spleen | 15.6 | 11.7 |
|  | Testes | 18.0 | 18.1 |
|  | Thyroid | 13.5 | 15.7 |

Expression levels detected for P1and P2 of the *HNFA* gene. The tissue-specific

expression patterns that match published data for P1 and P2 are shown in bold.
